# The Efficacy and Safety of Hepatic Arterial Infusion Chemotherapy Based on FOLFIRI for Advanced Intrahepatic Cholangiocarcinoma as Second-Line and Successive Treatment: A Real-World Study

**DOI:** 10.1155/2022/9680933

**Published:** 2022-09-26

**Authors:** Peixin Huang, Xiaoyong Huang, Yingting Zhou, Guohuan Yang, Qiman Sun, Guoming Shi, Yi Chen

**Affiliations:** ^1^Department of Hepatic Oncology, Liver Cancer Institute, Zhongshan Hospital, Fudan University, Shanghai, China; ^2^Department of Liver Surgery and Liver Transplantation, Liver Cancer Institute, Zhongshan Hospital, Fudan University, Shanghai, China

## Abstract

**Objective:**

Intrahepatic cholangiocarcinoma (iCCA) is a primary liver malignancy with a poor prognosis and limited treatment. Cisplatin with gemcitabine is used as the standard first-line chemotherapy regimen; however, there is still no robust evidence for second-line and successive treatments. Although preliminary evidence suggests a vital role of precision therapy or immunotherapy in a subset of patients, the gene alteration rate is relatively low. Herein, we explored the second-line and successive treatments using hepatic arterial infusion chemotherapy (HAIC) based on FOLFIRI after the failure of gemcitabine and platinum combined with target and immunotherapy in refractory CCAs.

**Methods:**

Advanced patients with iCCAs confirmed by diagnostic pathology, who progressed at least on a gemcitabine/platinum doublet and/or other systemic chemotherapy combined with target therapy and immune checkpoint inhibitor, were included. All patients received infusional 5-fluorouracil/leucovorin with irinotecan (FOLFIRI) via HAIC until progression or unacceptable toxicity. The primary objective was the feasibility of treatment, with secondary objectives of disease control rate (DCR) and 6-month survival rate.

**Results:**

A total of 9 iCCA patients treated between Dec 2020 and May 2021 were enrolled; 2 patients suffered from distant metastasis, while 7 had local lymph node metastasis and portal vein or hepatic vein invasion. HAIC was delivered as second-line therapy in 6/9 patients, while a third or successive therapy in 3/9 patients. The patients accepted an average of 2.90 ± 1.69 cycles of HAIC. The objective response rate was 22.2%; the disease control rate was 55.5% (5/9); median progression-free survival was 5 months; and 6-month survival rate was 66.7% (6/9).

**Conclusions:**

Our results provide preliminary evidence that HAIC based on FOLFIRI regimen is efficient and safe in some patients progressing after previous treatment. Therefore, HAIC may be a promising and valuable complementary therapy for advanced CCAs as a second-line and successive therapy. Otherwise, the combination of HAIC with precision medicine may improve clinical benefits (clinical registration number: 2021BAT4857).

## 1. Introduction

Cholangiocarcinoma (CCA) is a group of heterogeneous malignant tumors originating from the biliary tract epithelium, classified as intrahepatic or extrahepatic according to their anatomical location. According to the available data, some 10% CCAs are intrahepatic [1]. Histologically, more than 90% of CCAs are adenocarcinomas [2]. CCAs, which are generally aggressive, are diagnosed in advanced stages with a very poor prognosis. Surgery is the only potentially curative treatment for CCA; however, only 25% of patients are candidates for surgical resection [3]. The average 5-year overall survival (OS) for CCA is low, reaching only 13–21% in patients with early-stage disease who undergo successful resection due to high relapse rates (68–86%) [4, 5]. For patients with unresectable disease, chemotherapy with gemcitabine and cisplatin is considered the standard first-line treatment. This treatment approach has been associated with a median OS of <1 year and poor performance status (ECOG ≥ 2) as the strongest prognostic factor [6, 7]. 5-FU-based systemic regimens remain the current care standard for patients with evaluated progression after the frontline therapy, whose survival benefit is limited. Otherwise, the application of tumor genomic sequencing and the development of molecular therapeutic agents targeted at mutations have greatly expanded the treatment options for biliary tract tumors [8]. IDH1 (approximately 13% identified in CCAs), FGFR2 (about 20% detected), NTRK (<1%), TMB, MSI/MMR (2–3%), KRAS, TP53, BRCA 1/2 (about 1–7%), BRAF, CDKN2A/B, BAP1, PIK3CA, and HER2 are potential and promising mutation profiles, which may direct the CCA targeted therapy [9–11]. Based on the current data, an estimated 38.9% of all biliary tract cancer patients may harbor potentially targetable mutations [12]. However, there is still a large cohort of patients who are without gene mutations that can guide target treatment or fail to afford targeted agents due to economic reasons. Therefore, an alternative therapeutic strategy for these patients is urgently needed.

Over recent years, hepatic arterial infusion chemotherapy (HAIC) has been considered a promising treatment option for patients with large hepatocellular carcinoma (HCC). Growing evidence has indicated that infusion of HAIC sustaining local high-concentration of toxicity agents in tumors without embolization provided a significant survival benefit for patients with advanced HCC and was well tolerant [13, 14]. However, there are limited data on the efficacy of HAIC in intrahepatic cholangiocarcinoma. Herein, we explored the efficacy and safety of HAIC with the regimen of 5-fluorouracil (5-FU)/leucovorin with irinotecan at least as a second-line therapy in iCCA patients in a real-life setting.

## 2. Materials and Methods

### 2.1. Patients

A total of 9 patients treated in Zhongshan Hospital, Fudan University, Shanghai, China, between Dec 2020 and May 2021 were enrolled in the present study. Inclusion criteria were the following: (1) adults (≥18 years old) with histologically proven, unresectable iCCA; (2) those with a good performance status (ECOG 0–1); (3) those with radiologically proven progression on at least first-line chemotherapy (gemcitabine and platinum agent combined with an immune checkpoint inhibitor (PD-1 inhibitors) and/or lenvatinib, and (4) those with adequate liver function. Patients were excluded if they were medically unfit to receive chemotherapy.

Six to eight weeks after the first HAIC, all patients underwent a comprehensive evaluation, including hematologic examination and radiological assessment (enhanced abdomen MRI). Among all the enrolled patients, 6 received at least two courses of HAIC procedures, while the remaining 3 patients did not receive the subsequent HAIC treatment due to intolerant side effects and/or liver decompensation.

### 2.2. Hepatic Arterial Infusion Chemotherapy

Before HAIC procedure, all the patients were required to have adequate hematologic (leukocyte ≥ 4.0 × 10^9^/L, hemoglobin ≥ 90 g/L, and platelet count ≥ 80 × 10^9^/L) and hepatorenal function (serum total bilirubin level (TBil) ≤ 50.0 *μ*mol/L, serum alanine aminotransferase level (ALT) ≤ 80 U/L, and serum creatinine concentration ≤ 130 *μ*mol/L). The HAIC procedures were performed by experienced physicians. During the HAIC procedures, selective angiographies of the celiac trunk, superior mesenteric artery, and common hepatic artery were performed using a 4FRH catheter (Cordis, Miami Lakes, USA) via the right femoral artery approach or using a 4F MPA catheter (Cordis, Miami Lakes, USA) via left radical artery approach, alternatively. Superselection was done by microcatheter (Terumo, Tokyo, Japan) in all cases, and the tips of the microcatheter were ensured to localize the tumor feeding artery.

In the present study, we used 5-FU with irinotecan (FOLFIRI) regimen. Irinotecan (180 mg/m^2^ 30–90 min, d1) followed by leucovorin (LV, 400 mg/m^2^ given 2 h, d1) and fluorouracil (5-FU, 2400 mg/m^2^ maintain 44–46 h) were infused through the microcatheter on a 21-day cycle. During the maintenance of 5-FU, low molecule heparin was served (4100 U iH. qd) as an anticoagulant to prevent embolus formation. HAIC was continued until unacceptable toxicity or disease progression. Toxicity was recorded and evaluated by the National *Cancer* Institute Common Toxicity Criteria for Adverse Events (NCI-CTCAE), version 5.0.

### 2.3. Assessment and Statistical Analysis

Enhanced abdominal magnetic resonance imaging (MRI) was done for every patient at baseline and subsequently every 6 to 8 weeks after treatment. Efficacy was assessed by clinical physicians according to RECIST v1.1 criteria. Progression-free survival (PFS) was determined as the time from first HAIC to death or progression disease per RECIST 1.1 criteria. Overall survival was determined as the time from the first HAIC to death by any cause. Objective response rate (ORR); the proportion of patients with the best response of complete response (CR) or partial response (PR) according to RECIST 1.1) and disease control rate ((DCR) the proportion of patients with CR, PR, and stable disease (SD)) were also calculated. Once the patient was evaluated tumor progression, HAIC was stopped, and new antitumor therapy began. Categorical variables are described as frequencies and percentages and continuous variables as a median.

The study was performed in accordance with the Declaration of Helsinki and approved by the Clinical Research Ethics Committee of Fudan University Affiliated Zhongshan Hospital.

## 3. Results

### 3.1. Patient Characteristics

The patients were recruited over six months, and their characteristics are summarized in Table 1. Nine patients, 3 females and 6 males, with an average age of 55.3 years, were enrolled. Progression after gemcitabine plus oxaliplatin combined with lenvatinib and immune checkpoint inhibitors (PD-1 with different brands) as the first-line therapy was observed in 6 patients who then accepted HAIC based on FOLFIRI. Two patients accepted gemcitabine/oxaliplatin and lenvatinib/PD-1 as first-line therapy and failed by nanoparticle albumin-bound (NAB) paclitaxel plus gemcitabine as second-line therapy. One female patient took HAIC based on FOLFIRI as third-line therapy. In 2 patients, progression was observed after three-line treatment, and HAIC was adopted as a fourth-line therapy. Most patients had ECOG performance status 0–1, with two patients scoring 2 at entry. Seven patients were Child–Pugh class A (score 5–6), while 2 were Child–Pugh class B (score 7). At baseline, 2 patients suffered from distant metastasis, while 7 had local lymph node metastasis and portal vein or hepatic vein invasion.

### 3.2. Treatment Delivery

The average treatment cycle was 2.9 times (ranging from 1 to 5 cycles) per patient. Dose delay was seen in almost all cases that underwent at least two treatment cycles. There was no significant bone marrow suppression and liver function deterioration in patients with Child–Pugh stage A at entry; therefore, no patients required dose modification. Two patients with ECOG score 2 and Child–Pugh stage B at entry suffered liver failure after accepting HAIC as a fourth-line therapy. Only 1 patient (11.1%) ceased the HAIC treatment due to intolerance.

### 3.3. Efficacy

**Figure 1:**
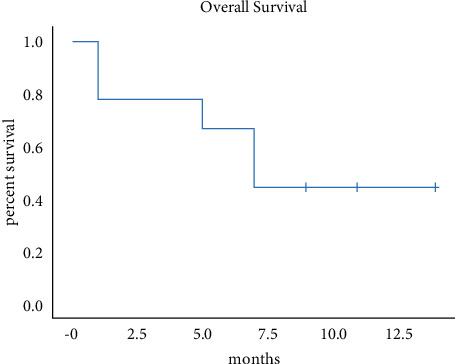
Kaplan–Meier estimates for the treated population.

**Figure 2:**
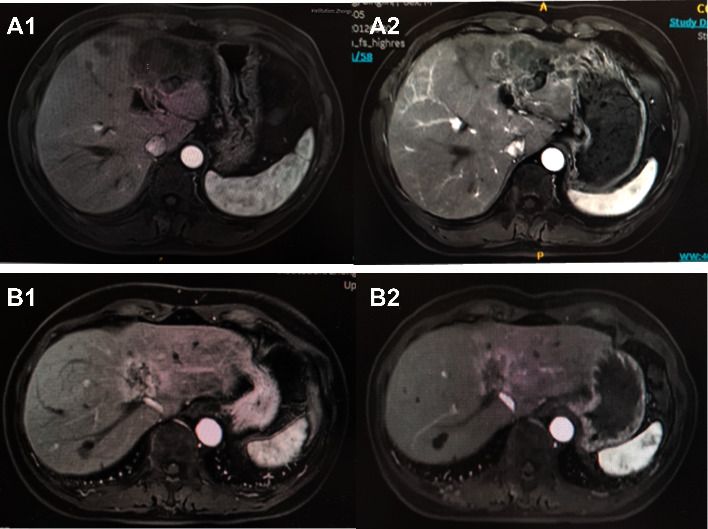
Imagine scans of two PR patients with best response of PR. (a) Tumor size of Patient No. 1 at baseline; (b) tumor in the left lobe had shrunk at first imagine assessment; (c) tumor size of Patient No. 3 at baseline; and (d) tumor had partial regression at first imagine assessment.

According to RECIST 1.1, the best response to HAIC based FOLFIRI regimen as a second-line and successive therapy in our unresectable iCCA cohort was PR in two patients, with an objective response rate (ORR) of 22.2%. The disease was stabilized in three patients (33.3%). Therefore, a disease control rate (DCR) of 55.5% was achieved. The tumor progression was found in two patients at the first imaging evaluation. The follow-up ended on November 10, 2021. Median PFS was 5.0 months (95% CI: 2.65–7.34), and median OS was 8.0 months (95% CI 6.04–9.96). Six-month survival rate was 66.7%. The concrete data are listed in Table 2. The Kaplan–Meier estimates for the treated population are shown in Figure 1. Imagine scans of two PR patients are shown in Figure 2.

### 3.4. Safety

Grade 3–4 toxicity was observed in two patients who accepted HAIC based on FOLFIRI regimen as a fourth-line therapy. Rapid deterioration of liver function, including progressively increased hyperbilirubinemia, elevated ALT and AST, and refractory ascites that finally resulted in death, occurred in 2 patients. Other common side effects included nausea (88.9%), vomiting (77.8%), hair loss (11.1%), oral ulceration (11.1%), and radical artery embolus (11.1%). One female patient suffered from radical artery embolus after HAIC via a radical artery. The incidence of AEs is listed in Table 3. One patient refused the subsequent HAIC due to Grade 3 nausea and vomiting.

## 4. Discussion

Intrahepatic cholangiocarcinoma (iCCA), which represents <10% of all CCA cases and is often diagnosed at an advanced disease stage, is associated with poor prognosis and limited life expectancy. For patients without surgical indications, systemic treatment is the first choice. ABC-02 phase 2 trial established the gemcitabine with cisplatin as the standard frontline therapy for CCA, with a response rate of 81.4% and median OS of 11.7 months [15]. Several other combination chemotherapy regimens have also been investigated, achieving response rates of 25% with gemcitabine/capecitabine, 50% with gemcitabine/oxaliplatin, 30% with gemcitabine/nab-paclitaxel, and 84% with nab-paclitaxel plus gemcitabine/cisplatin.

A 5-FU-based systemic regimen is currently considered the standard of care after progression on the frontline therapy. The phase 3 ABC-06 trial showed the improvement in OS following 5-FU/oxaliplatin with active symptom control compared to active symptom control alone (median OS 6.2 months vs. 5.3 months) [16]. Data for 5-FU with irinotecan (FOLFIRI) in the second-line setting in CCAs are limited. Recently, the Korean NIFTY study showed improved PFS and OS with liposomal irinotecan with 5-FU/leucovorin compared with 5-FU/leucovorin alone after progression on gemcitabine/cisplatin (PFS 7.1 month vs. 1.4 months, OS 8.6 months vs. 5.5 months) [17]. A small cohort retrospective study reported a median OS of 5 months in 12 unresectable CCA patients, indicating the beneficial effect on survival following FOLFIRI regimen as second-line therapy in CCA [18].

With the advent of tumor next-generation genomic sequencing and the development of targeted therapeutics directed at mutations at the molecular level, the treatment arsenal for biliary tract tumors tends to be more diversified and more individualized. According to several completed trials, FDA approved agents targeting IDH1 and FGFR2 for bile duct cancers progressing after standard chemotherapy [19, 20]. The phase 3 ClarIDHy trial showed that ivosidenib in CCA patients with an IDH1 mutation who progressed after 2 prior chemotherapies derived a PFS benefit of 6.9 months and median OS of 10.8 months compared with 1.4 months and 9.7 months in the placebo group [19]. Moreover, a phase 2 FIGHT-202 trial demonstrated that pemigatinib had a 35.5% objective response rate in iCCA patients with FGFR2 fusion/rearrangements in the second-line setting. In these patients, a PFS of 6.9 months and median OS of 21.1 months were observed [20]. Futibatinib (TS-120), an FGFR inhibitor, led to an ORR of 37.7%, DCR of 82%, and median PFS of 7.2 months in advanced CCA patients who progressed after at least one prior line of therapy and with FGFR2 fusions/rearrangements (FOENIX-CCA2 trial) [21]. Infigratinib (BGJ398), an FGFR inhibitor, led to an ORR of 23.1%, median PFS of 7.3 months, and median OS of 12.2 months in patients with FGFRS fusions/rearrangements progressed after the frontline therapy [22]. NTRK fusion-directed therapy is also considered a standard treatment option for eligible patients. According to the results of several clinical trials, entrectinib and larotrectinib were found to have a 57% and 75–80% response rate in advanced or metastatic cancer patients with NTRK fusion [23, 24]. The use of MET inhibitor in combination with gemcitabine achieved a 46% of SD and 20% of PR in patients with MET overexpression (about 34% seen in biliary tract cancers) in a small phase 1 trail [4]. Many other targeted therapies have also been investigated or are currently under investigation for use in CCA [25, 26]. CCA patients with dMMR tumors or high TMB could also be suitable candidates for immunotherapy [11, 27, 28].

Although the significant clinical benefit for these patients with tumors harboring gene alteration is significant and supports the wide testing of all patients for this molecular marker, the mutation rate is relatively low compared to the large population of CCA patients. Otherwise, a substantial portion of patients in China fail to afford targeted agents due to economic reasons. An alternative treatment is of urgent importance for patients without therapeutic gene mutation, progression after standard of care, and those unable to afford targeted therapy.

Local therapy such as transarterial radioembolization (TARE) or yttrium-90 (Y-90) treatment for iCCA was occasionally reported, achieving disease control with a median OS of 15.5 months with radiographic 28% of PR and 54% SD [29]. FOLFOX-HAIC significantly improved overall survival over TACE in patients with unresectable large hepatocellular carcinoma [13]. Nevertheless, hepatic arterial infusion of chemotherapy in iCCA has been rarely studied. A small phase 2 trial conducted by Cercek et al. showed that among 12 patients who accepted floxuridine via HAIC in combination with systemic gemcitabine and oxaliplatin as first-line therapy, disease control at 6 months was achieved in 84% of them, with a median OS of 25 months [30]. This small sample study suggested the feasibility of HAIC in iCCA treatment. A recent meta-analysis (pooling data from 9 studies that had enrolled a total of 154 patients) showed that hepatic arterial infusion pump (HAIP) chemotherapy with floxuridine for patients with unresectable iCCA was associated with a favorable 3-year OS of 39.5% (95% CI 31.5–47.4%) compared with systemic chemotherapy, with no patients surviving beyond 3 years observed in the ABC trial [31]. Therefore, we further explored the application of HAIC based on the FOLFIRI regimen in iCCA patients as a second-line and successive therapy.

In the present study, 9 patients were enrolled and accepted an average of 2.9 cycles (average, range 1–5 cycles) of HAIC procedures. Six patients accepted HAIC as a second-line treatment, while 3 patients accepted HAIC as a third-line and successive treatments. There were two patients for whom a partial response was evaluated as the best response according to RECIST 1.1 criteria, and 3 who achieved SD. Two patients died before the first imaging assessment. The possible reasons may be the late stage of disease when accepting HAIC as a fourth-line treatment and deterioration of liver function associated with the tumor. We achieved an objective response rate (ORR) of 22.2% and a disease control rate (DCR) of 55.5%. Median PFS and median OS were 5.0 months (95% CI:2.65–7.34) and 8.0 months (95% CI 6.04–9.96), respectively. The six month survival rate was 66.7% till Nov 10th, 2021. Our results showed that HAIC based on FOLFIRI was to some extent efficient in iCCA patients as second line, but not to late stage treatment. The prolonged interval of HAIC may be the factor affecting efficacy. In this study, the average interval between two HAIC procedures was 37.7 ± 36 days, which is significantly longer than the standard of 21 days. The deferred HAIC mostly occurred after three times HAIC or after the first imagine assessment. Patients evaluated prolonged HAIC as uncomfortable bedridden experiences, especially continuous HAIC via the femoral artery. The prolonged interval may affect the cytotoxicity of chemical agents to the tumor, therefore impairing the antitumor effects. HAIC via radical artery could improve the comfort of patients. However, since the radical artery is relatively thin and the artery catheter needs to be placed for more than 46 hours, the incidence of local swelling and embolus may increase. Future studies should focus on the optimization of HAIC procedures and chemotherapy duration. The limitation of the present study is the small sample size that does not reflect its generalizability, therefore this study must be considered preliminary and exploratory and should be confirmed by other studies. An expanded sample size is needed to further validate our findings to obtain more evidence for the survival benefit of this therapy.

Most patients could tolerate HAIC treatment well. The most commonly seen AEs were gastrointestinal adverse events, including nausea and vomiting, which were correlated with chemotherapeutics. One patient stopped the subsequent HAIC procedure due to Grade 3 vomiting (11.1%). The respective frequency of hair loss, oral ulceration, weight loss, and local embolus was 22.2%, 11.1%, 11.1%, and 11.1%, resulting in the severity of Grade 1–2. One patient suffered from the left radical artery embolus with no symptoms, perhaps due to a relatively small radical artery. The patient rapidly recovered after anticoagulant treatment. Grade 1–2 hyperbilirubinemia and elevated ALT and AST were seen in 33.3%, 22.2%, and 22.2% of patients and were recovered within one week after HAIC. Grade 3–5 hyperbilirubinemia, elevated ALT and AST, and refractory ascites occurred in 2 patients who died one month after HAIC. Their death was considered to be correlated with the late stage of disease and rapid tumor progression, but probably not with HAIC per se. Therefore, the HAIC may not be suitable for patients with poor physical condition and accelerating tumor progression. The HAIC may not be applied as a third-line or successive treatment. Active symptom control support should be optimized and emphasized.

## 5. Conclusions

In summary, the present study provides preliminary evidence that FOLFIRI-HAIC exhibits partial efficacy and safety in unresectable intrahepatic cholangiocarcinoma patients with disease progression after frontline treatment, irrespective of gene mutation. As this was a small cohort feasibility real-world study, our results are promising. The FOLFIRI-HAIC regimen could be an alternative and complementary option for patients without gene mutation, patients who failed prior therapy, and patients who failed to achieve target therapy. Yet, a larger sample study is required to further evaluate the outcomes following this treatment strategy. Since most patients were well tolerant to HAIC, the HAIC-FOLFIRI model could also be considered in combination with the target agent to achieve better outcomes in patients eligible for intensive treatment.

## Figures and Tables

**Table 1:** The summary of patient characteristics.

Charateristics		N
	Age	53 (median, range: 39–68)
	Gender	
	Female	3 (33.3%)
	Male	6 (66.7%)
	ECOG	
	0	5 (55.6%)
	1	2 (22.2%)
	2	2 (22.2%)
	Child–Pugh	
	A	7 (77.8%)
	B	2 (22.2%)
	C	0
	Max tumor size (cm)	70.7 ± 30.0 (average, range 33–130)
	Prior lines of treatment	
	1	6 (66.7%)
	2	1 (11.1%)
	3	2 (22.2%)
	Previous treatment regimen	
1st line	Gemox + levatinib + PD-1	7
	GP + PD-1	2
2st line	capecitabine + nanoparticle albumin-bound (NAB) paclitaxel	2
	FGFR2 inhibitor (clinical trial)	1
3st line	Gemox + Levatinib + PD-1 inhibitor	2
	CA19-9 at baseline	
	Positive	5 (55.6%)
	Negtive	4 (44.4%)
	HAIC cycles	2.9 (average, range 1–5)

ECOG: the Eastern Cooperative Oncology Group performance status; Gemox: oxaliplatin plus gemcitabine regimen; GP: gemcitabine plus cisplatin regimen; CA19-9 positive: serum CA19-9 expression above 34 U/L; and PD-1 inhibitors: patients chose different PD-1 inhibitors including pembrolizumab, tislelizumab, and Sintilimab.

**Table 2:** The summary of treatment.

Patients	Cycles of treatment (*N*)	Best response	Progression-free survival (months)	Overall survival (months)	Surival status
1	5	PR	5	7	Death
2	1	SD	3	5	Death
3	5	PR	6	13	Alive
4	4	SD	6	7	Alive
5	4	SD	5	14	Alive
6	1	-	1	1	Death
7	3	PD	3	9	Alive
8	1	-	1	1	Death
9	2	PD	2	9	Alive

PR: partial response; SD: stable disease; and PD: progression disease.

**Table 3:** Adverse events and frequency.

AEs	Grade 1–2	Frequency (%)	Grade 3–4	Frequency (%)	Total frequency (%)
Nausea	7	77.8	0	0.0	77.8
Vomiting	6	66.7	1	11.1	77.8
Hair loss	2	22.2	0	0.0	22.2
Oral ulceration	1	11.1	0	0.0	11.1
Hyperbilirubinemia	3	33.3	2	22.2	55.6
Elevated ALT	2	22.2	2	22.2	44.4
Elevated AST	2	22.2	2	22.2	44.4
Ascite	0	0.0	2	22.2	22.2
Weight loss	1	11.1	0	0.0	11.1
Embolus	1	11.1	0	0.0	11.1

## Data Availability

The datasets used in supporting this article are available from the corresponding authors on reasonable request.
